# MDM2–GPX4–ferroptosis regulatory axis exerts neurotoxic effects in intracerebral hemorrhage

**DOI:** 10.4103/NRR.NRR-D-25-00030

**Published:** 2025-08-13

**Authors:** Yunhu Yu, Tao Liu, Yunpeng Cai, Yuanmei Song, Hang Zhou, Fang Cao, Rongcai Jiang

**Affiliations:** 1 Department of Neurosurgery, Tianjin Neurological Institute, State Key Laboratory of Experimental Hematology, Laboratory of Post‐Neuroinjury Neurorepair and Regeneration in Central Nervous System Tianjin & Ministry of Education, Tianjin Medical University General Hospital, Tianjin, China; 2 Department of Neurosurgery, Xuanwu Hospital, Capital Medical University, Beijing, China; 3 Department of Neurosurgery, South China Hospital of Shenzhen University, Shenzhen, Guangdong Province, China; 4 Department of Neurosurgery, the People’s Hospital of HongHuaGang District of ZunYi, Zunyi, Guizhou Province, China; 5 Department of Neurosurgery, Affiliated Hospital of Zunyi Medical University, Zunyi, Guizhou Province, China

**Keywords:** ferroptosis, glutathione peroxidase 4, intracerebral hemorrhage, m6A, murine double minute 2, ubiquitination

## Abstract

Ferroptosis plays a key role in nerve injury in intracerebral hemorrhage and is associated with the upregulation of murine double minute 2. Investigating the mechanism underlying murine double minute 2-related ferroptosis could help identify new therapies for intracerebral hemorrhage. An *in vitro* intracerebral hemorrhage model was established by treating BV2 microglial cells with oxygen–glucose deprivation combined with hemin. The role of murine double minute 2 in regulating ferroptosis was investigated via transduction with RNA interference and lentivirus overexpression. Furthermore, intracerebral hemorrhage mouse models were constructed with and without an murine double minute 2 inhibitor (brigimadlin), and behavioral assays were performed to assess the learning ability and cognitive function. Murine double minute 2 dysregulation was associated with oxygen–glucose deprivation combined with hemin-induced BV2 microglial cell ferroptosis and M1/M2 polarization. The results suggested that murine double minute 2 induced glutathione peroxidase 4 ubiquitination and degradation to regulate ferroptosis and inflammatory responses in BV2 microglial cells. Mechanistically, Wilms tumor 1-associated protein induced murine double minute 2 N6-methyladenosine (m6A) modification and regulated ferroptosis and inflammatory responses. *In vivo* analysis showed that brigimadlin improved neurological deficits and spatial memory in mice with intracerebral hemorrhage. In summary, the results indicate that Wilms tumor 1-associated protein regulates murine double minute 2 m6A modification, and murine double minute 2 induces glutathione peroxidase 4 ubiquitination and degradation. This regulation promotes ferroptosis and inflammatory responses in oxygen–glucose deprivation combined with hemin-induced BV2 microglial cells, suggesting that the murine double minute 2–glutathione peroxidase 4–ferroptosis regulatory axis exerts neurotoxic effects. These findings identify glutathione peroxidase 4 as a potential gene therapy target for intracerebral hemorrhage–related brain injury.

## Graphical abstract

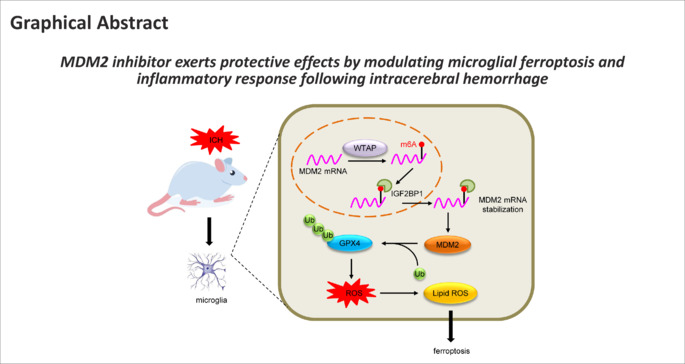

## Introduction

Intracerebral hemorrhage (ICH), also called spontaneous cerebral hemorrhage, is caused by ruptured brain blood vessels and accounts for 10%–15% of all strokes (Kung et al., 2024). Current ICH treatments are primarily divided into internal medicine and surgical treatments. Critically ill patients or those with secondary causes must undergo surgery. In addition, corresponding medications are required to address complications, such as high intracranial pressure and hypertension, caused by ICH (Ziai and Carhuapoma, 2018). ICH is characterized by high mortality and morbidity, and its mechanisms, which are responsible for extensive neurological damage, are not fully understood (Kanamaru and Suzuki, 2025; Ning et al., 2025). Increasing evidence suggests that microglia are the initial nonneuronal responders to various acute brain injuries (Fei et al., 2024; Lu et al., 2024). Investigating microglial responses to brain injuries and post-ICH cell death patterns is crucial to better understand ICH mechanisms.

Ferroptosis—an iron- and lipid peroxidation-dependent cell death—is involved in multiple central nervous system disorders (Weiland et al., 2019; Fei et al., 2024). Its primary mechanism involving iron or ester oxygenase facilitates high expression of unsaturated fatty acids on cell membranes, resulting in lipid peroxidation and reactive oxygen species (ROS) production, ultimately inducing cell death (Liang et al., 2023). Another mechanism is the reduction of the crucial antioxidant enzyme glutathione peroxidase 4 (GPX4), which regulates antioxidant pathways (Miao et al., 2022). GPX4, which is located in mitochondria, cytoplasm, and nuclei, uses glutathione (GSH) to reduce lipid peroxides to normal phospholipid molecules, preventing ferroptosis (Seibt et al., 2019). Research of ferroptosis in microglia after ICH is still in the preliminary stage, and the mechanisms underlying microglial responses to ferroptosis remain unclear.

Murine double minute 2 (MDM2) is a crucial cellular regulatory protein associated with poor cancer prognosis (Iwakuma and Lozano, 2003) that influences many cellular biological processes, including growth, angiogenesis, metabolism, apoptosis, and metastasis (Zafar et al., 2023). MDM2 levels increase following ICH and cerebral ischemia and contribute to the apoptosis of neuronal and brain microvascular endothelial cells (Xu et al., 2018; Guo et al., 2021). Moreover, MDM2 is the master regulator of p53 stabilization and contributes to ferroptosis. MDM2 induces ferroptosis via p53-dependent or p53-independent mechanisms (Han et al., 2024; Ji et al., 2024). MDM2 also promotes *Staphylococcus aureus*-induced ferroptosis in osteomyelitis via the TLR4/SLC7A11/GPX4 signaling pathway (Song et al., 2024). However, whether MDM2 regulates ferroptosis in an ICH model remains unclear.

N6-methyladenosine (m6A) is an epigenetic RNA modification that regulates gene expression by adding a chemical marker to RNA molecules. This dynamic and reversible process regulates RNA stability, splicing, processing, transcription, and translation (Jiang et al., 2021). Wilms tumor 1-associated protein (WTAP) is a methyltransferase that interacts with the N-terminal helix of methyltransferase-like 3 (METTL3) via its N-terminal helical coil region to localize METTL3/14 to the nucleus, facilitating m6A modification (Liu et al., 2023). Furthermore, the m6A modification of MDM2 regulates ferroptosis in various cancers, organ injuries, and inflammatory conditions (Mu et al., 2022; Hu et al., 2023; Han et al., 2024; Song et al., 2024). Therefore, we hypothesized that MDM2 m6A modifications promote microglial ferroptosis after ICH.

This study investigated the effects of MDM2 on ferroptosis during ICH. We hypothesized that the m6A modification regulates MDM2 to promote ICH progression by inducing ferroptosis.

## Methods

### Cell culture

Mouse BV2 microglial cells (Sunncell, Wuhan, Hubei, China, RRID: CVCL_0182) were subjected to oxygen and glucose deprivation combined with hemin (OGD/H) to develop an *in vitro* ICH model (Chen et al., 2021). In brief, BV2 microglial cells were cultured in glucose- and serum-free Dulbecco’s modified Eagle medium (Gibco, Carlsbad, CA, USA) under 95% N_2_ and 5% CO_2_ for 10 minutes. Cells were subsequently treated with 10 μM hemin (Sigma-Aldrich, Merck, Darmstadt, Germany) for 120 minutes under 95% N_2_ and 5% CO_2_, and then were restored to normal culture conditions. BV2 microglial cells were pretransduced for 24 hours with MDM2 shRNA, GPX4 shRNA, WTAP shRNA, scramble shRNA (shNC), MDM2-overexpression, (oeMDM2) or blank lentivirus vector (all from Genepharma, Shanghai, China), and were subsequently subjected to OGD/H for 24 hours. To inhibit ferroptosis, apoptosis, or necroptosis, cells were treated with OGD/H or pretransduced with the oeMDM2 or blank lentivirus vector for 24 hours, followed by treatment with 1 μM ferrostatin-1 (Fer-1), 20 μM Z-VAD-FMK, or 20 μM necrostatin-1 (all from Selleck, Shanghai, China), respectively, for 24 hours. To inhibit protein degradation, cells were pretransduced with the oeMDM2 or blank lentivirus vector for 24 hours, followed by 10 μM MG132 (Selleck) treatment for 4 hours.

### Construction of lentiviral cell lines

The pLVX-Puro lentivirus vector was used to construct the oeMDM2 vector. pLKO.1-MDM2-shRNA fragments 1 and 2 (shMDM2-1 and shMDM2-2, respectively), pLKO.1-GPX4-shRNA fragments 1 and 2 (shGPX4-1 and shGPX4-2, respectively), pLKO.1-WTAP-shRNA fragments 1 and 2 (shWTAP-1 and shWTAP-2, respectively), and pLKO.1-insulin-like growth factor 2 mRNA binding protein 1 (IGF2BP1)-shRNA fragments 1 and 2 (shIGF2BP1-1 and shIGF2BP1-2, respectively) lentivirus vectors were used to construct MDM2, GPX4, WTAP, and IGF2BP1 knockdown vectors, respectively. Blank control pLVX-Puro, oeMDM2, corresponding pLKO.1-shRNA fragments, and shNC lentivirus vectors together with pMD2G and psPAX2 packaging plasmids were cotransfected into HEK-293T cells (RRID: CVCL_0063) using Lipofectamine 2000 (Invitrogen, Thermo Fisher Scientific, Waltham, MA, USA). After 48 hours, lentivirus vectors were harvested and used to transduce BV2 microglial cells (Geng et al., 2024). Different shRNAs were purchased from Shanghai Genechem Co., Ltd. (Shanghai, China) and shown in **Additional Table 1**.

**Additional Table 1:** shRNA sequences used in the study

shRNA fragments	Sequence (5’-3’)
MDM2 shRNA fragment 1 (shMDM2-1)	GACAGAGAATGATGCTAAA
MDM2 shRNA fragment 2 (shMDM2-2)	CACATTGTGTATTGTTCAA
WTAP shRNA fragment 1 (shWTAP-1)	GACAGAGAATGATGCTAAA
WTAP shRNA fragment 2 (shWTAP-2)	CACATTGTGTATTGTTCAA
GPX4 shRNA fragment 1 (shGPX4-1)	GAAGTAATCAAGAAATCAA
GPX4 shRNA fragment 2 (shGPX4-2)	AGTTTGACATGTACAGCAA
IGF2BP1 shRNA fragment 1 (shIGF2BP1-1)	CGATGGGAAGTGCTAGATA
IGF2BP1 shRNA fragment 2 (shIGF2BP1-2)	CAAGCTATCATGAAGCTAA

### Cell Counting Kit-8 assay

BV2 microglial cells were maintained overnight at 37°C. After treatment, 10 μL Cell Counting Kit-8 reagent (Beyotime Biotechnology, Shanghai, China, Cat# C0039) was added and the cells were subsequently incubated for 1 hour. Cell viability was determined by measuring the optical density value at 450 nm using an enzyme labeler (Epoch2, Bio Tek Epoch, Santa Clara, CA, USA).

### Lactate dehydrogenase assay

Lactate dehydrogenase (LDH) activity in BV2 microglial cells was assessed using an LDH assay kit (Nanjing Jiancheng Bioengineering Institute, Nanjing, China, Cat# A020-2) according to the manufacturer’s instructions.

### Fe^2+^, glutathione, and malondialdehyde level detection

Fe^2+^ levels in BV2 microglial cells and mouse brain tissue were quantified using an iron colorimetric assay kit (Abcam, Waltham, MA, USA, Cat# ab83366). GSH (Solarbio Science & Technology, Beijing, China, Cat# BC1175) and malondialdehyde (MDA; Solarbio Science & Technology, Cat# BC0025) concentrations in BV2 microglial cells and mouse brain tissue were assessed using their respective assay kits.

### Lipid reactive oxygen species measurement

C11-BODIPY probe was used to measure lipid ROS in BV2 microglial cells. Briefly, C11-BODIPY (Thermo Fisher Scientific) was added to the cell suspension and incubated for 30 minutes at 37°C. The samples were washed with phosphate buffered saline, and fluorescence intensity of the samples was measured using a CytoFLEX flow cytometer (Beckman Coulter, Fullerton, CA, USA).

### Quantitative reverse transcription-polymerase chain reaction

Total RNA was isolated from BV2 microglial cells and mouse brain tissue using TRIzol reagent (Thermo Fisher Scientific). Reverse transcription was carried out using a PrimeScript kit (Takara Bio Inc, Shiga, Japan). Levels of the target genes were analyzed by quantitative reverse transcription-polymerase chain reaction (qRT-PCR) using SYBR green PCR master mix (Applied Biosystems, Foster City, CA, USA), according to the manufacturer’s instructions. The primer sequences used for PCR are shown in **Additional Table 2**. The relative expression ratio of the target genes was normalized to β-actin gene expression using the ΔCt method (2^–ΔΔCt^). The PCR cycling conditions were 95°C for 5 minutes, followed by 40 cycles of 95°C for 10 seconds, 60°C for 30 seconds, and 78°C for 20 seconds.

**Additional Table 2:** The primers used for quantitative reverse transcription-polymerase chain reaction analysis

Gene	Primer sequences
MDM2	F: 5ʹ-TCAGGATCTTGACGATGGCG-3ʹ
	R: 5ʹ-ACTGTGACCCGATAGACCTCA-3ʹ
iNOS	F: 5ʹ-TGAAGAAAACCCCTTGTGCTG-3ʹ
	R: 5ʹ-TGCAAGTGAAATCCGATGTGG-3ʹ
IL-1	F: 5ʹ-ATGCCACCTTTTGACAGTGATG-3ʹ
	R: 5ʹ-TGATGTGCTGCTGCGAGATT-3ʹ
TNF-α	F: 5ʹ-TAGCCCACGTCGTAGCAAAC-3ʹ
	R: 5ʹ-GCAGCCTTGTCCCTTGAAGA-3ʹ
Arg-1	F: 5ʹ-GTACATTGGCTTGCGAGACG-3ʹ
	R: 5ʹ-ATCGGCCTTTTCTTCCTTCCC-3ʹ
IL-10	F: 5ʹ-GTAGAAGTGATGCCCCAGGC-3ʹ
	R: 5ʹ-TAGACACCTTGGTCTTGGAGC-3ʹ
TGF-β	F: 5ʹ-CTGCTGACCCCCACTGATAC-3ʹ
	R: 5ʹ-GGGGCTGATCCCGTTGATT-3ʹ
METTL3	F: 5ʹ-CGTAGTGATAGTCCCGTGCC-3ʹ
	R: 5ʹ-TGGCGTAGAGATGGCAAGAC-3ʹ
METTL14	F: 5ʹ-TATGCTTGCGAAAGTGGGGT-3ʹ
	R: 5ʹ-AATGAAGTCCCCGTCTGTGC-3ʹ
WTAP	F: 5ʹ-TGCAAGAGTGCACCACTCAA-3ʹ
	R: 5ʹ-TCAGGCGTAAACTTCCAGGC-3ʹ
IGF2BP1	F: 5ʹ-CTCCGGAGCAGGAGATGGTA-3ʹ
	R: 5ʹ-TTCTTCCCTGGGCCTTGAAC-3ʹ
β-actin	F: 5ʹ-GCGTGACATCAAAGAGAAGC-3ʹ
	R: 5ʹ-ATGCCACAGGATTCCATACC-3ʹ

Arg-1: Arginase 1; IGF2BP1: insulin-like growth factor 2 mRNA binding protein 1; IL: interleukin; iNOS: inducible nitric oxide synthase; MDM2: murine double minute 2; METTL: methyltransferase-like; TGF-β: transforming growth factor-β; TNF-α: tumor necrosis factor-α; WTAP: Wilms tumor 1-associated protein.

### Immunoblotting

Immunoblotting was conducted as described previously (He et al., 2021). BV2 microglial cells and mouse brain tissue were lysed on ice using radioimmunoprecipitation assay buffer, followed by centrifugation (12,000 × *g*, 20 minutes, 4°C). Proteins (25 µg) were separated by 10% sodium dodecyl sulfate-polyacrylamide gel electrophoresis, transferred to a nitrocellulose membrane (Millipore, Merck, Darmstadt, Germany), blocked in 5% fat-free milk (Elabscience Biotechnology, Wuhan, China) overnight at 4°C, and incubated overnight at 4°C with the following primary antibodies: anti-MDM2 (rabbit, 1:1000, Abcam, Cat# ab260074, RRID: AB_2936956), anti-GPX4 (rabbit, 1:1000, Abcam, Cat# ab125066, RRID: AB_10973901), anti-METTL3 (rabbit, 1:1000, Abcam, Cat# ab195352, RRID: AB_2721254), anti-METTL14 (rabbit, 1:1000, Abcam, Cat# ab220031, RRID: AB_2893210), anti-WTAP (rabbit, 1:500, Abcam, Cat# ab195380, RRID: AB_2868572), anti-IGF2BP1 (rabbit, 1:1000, Abcam, Cat# ab100999, RRID: AB_10865721), and anti-β-actin antibody (rabbit, 1:5000, Proteintech, Rosemont, IL, USA, Cat# 81115-1-RR, RRID: AB_2923704). The membranes were subsequently incubated with horseradish peroxidase-conjugated anti-rabbit secondary antibody (goat, 1:5000, ZSGB-BIO, Beijing, China, Cat# ZB-2301, RRID: AB_2747412) for 1 hour at 37°C. Protein bands were visualized using an ECL Detection Kit (Millipore, WBULP-100ML). The relative band densities were determined using ImageJ software (version 1.8.0, Media Cybernetics, Rockville, MD, USA). β-Actin was used as the internal reference to evaluate protein expression levels.

### Enzyme-linked immunosorbent assay

Tumor necrosis factor-α (TNF-α; Cat# E-EL-M3063), transforming growth factor- (TGF-; Cat# E-UNEL-M0099), interleukin-1 (IL-1; Cat# E-EL-M0037), and IL-10 (Cat# E-MSEL-M0031) levels in BV2 microglial cell supernatant and mouse brain tissue were measured using an enzyme-linked immunosorbent assay kit (all from Elabscience Biotechnology Co., Ltd.).

### Coimmunoprecipitation and ubiquitination analysis

BV2 microglial cellular lysate was prepared using immunoprecipitation (IP) buffer and was subjected to IP with primary antibodies against MDM2 (rabbit, 1:30, Abcam, Cat# ab259265, RRID: AB_2920616), GPX4 (rabbit, 1:30, Proteintech, Cat# 30388-1-AP, RRID: AB_3086304), or control immunoglobin G (IgG; 1:50, rabbit, Santa Cruz Biotechnology, Santa Cruz, CA, USA, Cat# sc-66931, RRID: AB_1125055) overnight at 4°C. The immunoprecipitated complexes were subsequently affinity-purified using protein A/G agarose beads (Santa Cruz Biotechnology). For analysis, the immunoprecipitated proteins were resolved via western blotting using primary antibodies against MDM2 (mouse, 1:1000, Abcam, Cat# ab16895, RRID: AB_2143534), GPX4 (mouse, 1:1000, Proteintech, Cat# 67763-1-Ig, RRID: AB_2909469) or ubiquitin (anti-Ub; rabbit, 1:1000, Abcam, Cat# ab134953, RRID: AB_2801561) overnight at 4°C, and then washed three times with Tris-buffered saline with 0.1% Tween-20 (Sigma-Aldrich, Merck). The membranes were subsequently incubated with horseradish peroxidase-conjugated anti-mouse secondary antibody (goat, 1:5000, ZSGB-BIO, Cat# ZB-2305, RRID: AB_2747415) or anti-rabbit secondary antibody (goat, 1:5000, ZSGB-BIO, Cat# ZB-2301, RRID: AB_2747412) for 1 hour at 37°C. β-Actin was used as the internal reference to evaluate protein expression levels. The membranes were then immersed in enhanced chemiluminescence solution (Millipore, WBULP-100ML) and visualized using an image-quantitative enhanced chemiluminescence imager (Millipore). The relative band densities were determined using ImageJ software (version 1.8.0, Media Cybernetics).

### Protein stability assay

To assess GPX4 protein turnover, BV2 microglial cells were pretransduced with the oeMDM2 or blank lentivirus vector for 24 hours, followed by cycloheximide (CHX; 0.1 mg/mL, Selleck) treatment for 0, 3, and 6 hours. Western blotting was performed using anti-GPX4 and anti-β-actin antibodies, and quantification was performed relative to β-actin using ImageJ software (version 1.8.0, Media Cybernetics).

### m6A content analysis

m6A content in BV2 microglial cells was assessed using an m6A RNA Methylation Assay Kit (Abcam, Cat# ab185912), according to the manufacturer’s instructions.

### m6A methylated RNA immunoprecipitation quantitative polymerase chain reaction

m6A methylated RNA immunoprecipitation quantitative polymerase chain reaction (PCR) (MeRIP-qPCR) was performed as previously described (Wang et al., 2023). Total RNA (100 µg) isolated from BV2 microglial cells subjected to OGD/H and transduced with shWTAP or shNC lentivirus vector was incubated overnight at 4°C with anti-m6A antibody (mouse, 1:30, Abcam, Cat# ab208577, RRID: AB_2916290) or anti-IgG antibody (rabbit, 1:50, Santa Cruz Biotechnology, Cat# sc-66931, RRID: AB_1125055) in RNA immunoprecipitation (RIP) buffer (Sigma-Aldrich, Merck) containing protease and RNase inhibitors (Thermo Fisher Scientific). RNA was subsequently eluted from the magnetic A/G beads (Sigma-Aldrich, Merck), and the m6A-modified MDM2 was quantified via qRT-PCR assay. The primer sequences were as follows: MDM2 3′ untranslated region (UTR) forward, 5′-CGA CTT CCA GCA GCA TTG-3′ and reverse, 5′-ACT GGG CAG GGC TTG TTT C-3′.

### Reporter gene assays

The MDM2 3′ UTR sequence was cloned into a pmirGLO vector (Promega, Madison, WI, USA). BV2 microglial cells subjected to OGD/H and transduced with shWTAP or shNC lentivirus vector were transfected with either the pmirGLO-MDM2 3′ UTR or pRL-TK vector using Lipofectamine 2000 (Thermo Fisher Scientific) as described previously (Li et al., 2025). Luciferase activities were gauged using the dual-luciferase reporter gene assay system (Promega).

### RNA stability

Actinomycin D (5 µg/mL; Sigma-Aldrich, Merck) was administered to evaluate RNA stability in BV2 microglial cells pretransduced with shIGF2BP1 or shNC lentivirus vector. Immediately after or following a 3- or 6-hour incubation, cell pellets were collected and subjected to RNA extraction, followed by qRT-PCR analysis.

### RNA immunoprecipitation assay

The RIP assay was performed using the RIP Kit (Sigma-Aldrich, Merck). Extracted RNA (100 µL) from BV2 microglial cells was incubated with RIP buffer containing magnetic A/G beads conjugated with anti-IGF2BP1 antibody (rabbit, 1:30, Proteintech, Cat# 22803-1-AP, RRID: AB_2879173). Anti-IgG antibody (rabbit, 1:50, Santa Cruz Biotechnology, Cat# sc-66931, RRID: AB_1125055) served as a negative control. RNA was subsequently eluted from the magnetic A/G beads, and the binding between IGF2BP1 and MDM2 3′ UTR was quantified usnig qRT-PCR assay.

### Establishment of an intracerebral hemorrhage mouse model

All animal experiments were approved by the Experimental Animal Welfare Ethics Committee of Tianjin Medical University General Hospital, Tianjin, China on June 3, 2025 (approval No. IRB2025-DW-57) and performed according to the National Institutes of Health Guidelines for the Care and Use of Laboratory Animals. To rule out the influence of sex on the results, only male mice were used (Wang et al., 2025). Specific pathogen free–grade male C57BL/6 mice (age, 8–10 weeks; weight, 25–30 g) were purchased from Vital River Laboratory Animal Technology Co., Ltd (Beijing, China, license No. SCXK (Jing) 2021-0006). The mice (three mice per cage) were kept in an animal room at 23–27°C, with humidity 50% ± 10%, and ad libitum access to food and water, under a standard 12/12-hour light/dark cycle. To construct the ICH mouse model, collagenase VII (0.5 μL dissolved in phosphate buffered saline, 0.075 U; Sigma-Aldrich, Merck) was stereotactically injected into the right basal ganglia (3.5 mm below the dura, 2.2 mm lateral to bregma, and 0.2 mm posterior to the coordinates) (Wu et al., 2022) with a 26-gauge needle via a microinfusion pump at a rate of 0.5 µL/min (Gong and Wei, 2025). After the model was established, mice were randomly assigned to either the intervention (ICH + brigimadlin) or vehicle group (ICH + vehicle). Brigimadlin (2.5 μL dissolved in DMSO, 10 mg/kg; Selleck) was injected into the right lateral ventricle 60 minutes after ICH via a microinfusion pump at a rate of 0.5 µL/min (Hao et al., 2023; Wang et al., 2025). Sham operations were conducted by needle insertion only. Mice that underwent sham surgery and those treated with vehicle were used as the controls. At 72 hours after ICH modeling, brain tissue from the ipsilateral side, 4 mm posterior to the injection site, was collected for hematoxylin and eosin (HE), dihydroethidium (DHE), and immunohistochemistry (IHC) staining. Tissue from the ipsilateral side, 3 mm anterior to the injection site, was collected for qRT-PCR, western blotting, enzyme-linked immunosorbent assay and biochemical analyses.

### Hematoxylin and eosin and immunohistochemistry stainings

Mouse brains were fixed in 4% paraformaldehyde for 48 hours, dehydrated in gradient ethanol, and cleared with xylene. Thereafter, brains were embedded in paraffin and sectioned (4 μm thickness). HE and IHC staining were conducted as previously reported (Zhi et al., 2024). HE staining was performed using an HE Staining Kit (Solarbio Science & Technology, Cat# G1120) according to standard histological protocols. For IHC staining, sections were incubated overnight with WTAP (mouse, 1:1000, Cat# 60188-1-Ig, RRID: AB_10859484), MDM2 (mouse, 1:200, Cat# 66511-1-Ig, RRID: AB_2881874), and GPX4 (mouse, 1:1000, Cat# 67763-1-Ig, RRID: AB_2909469; all from Proteintech) antibodies at 4°C. The sections were then incubated with horseradish peroxidase-conjugated anti-mouse IgG secondary antibody (goat, 1:500, Abcam, Cat# ab6789, RRID: AB_955439) for 30 minutes at 25°C. Immunoreactivity was visualized using dimethylbenzidine (Beyotime Biotechnology), and sections were counterstained with hematoxylin. Images were captured using a BX43 light microscope (Olympus, Tokyo, Japan) and the results were quantified in five randomly selected fields per section using Image-Pro Plus 6.0 (Media Cybernetics).

### Dihydroethidium staining

DHE solution (10 μM, Solarbio Science & Technology) was used to detect ROS production in fresh frozen mouse brain sections (10 μm thickness) as described previously (Chan et al., 2007). The fluorescence intensity was observed using a BX-61 fluorescence microscope (Olympus) and the results were quantified in five randomly selected fields per section using Image-Pro Plus 6.0 (Media Cybernetics).

### Neurological deficit score

Neurological deficits in mice were evaluated using the 28-point test (He et al., 2020) at 1, 3 and 7 days after ICH. The 28-point scale measures whisker response, compulsory circling, front limb symmetry, circling behavior, climbing, gait, and body symmetry. Each item is graded from 0 to 4, and total scores range from 0 to 28, with 0 indicating normal function and 28 representing the most severe deficit.

### Morris water maze test

At 7 days after ICH modeling, the Morris water maze test was used to test the spatial learning and memory abilities, as previously reported (Ashrafpour et al., 2024). The Morris water maze test comprised 5-day experimental trials conducted in the afternoon for all animals. Training trials (four per day) were conducted over 4 consecutive days. Escape latency was assessed to determine spatial learning. To assess spatial memory, the time animals spent in the target quadrant was quantified.

### Lactate dehydrogenase release cell death assay

Cell death rate was determined using the LDH cytotoxicity assay kit (Beyotime Biotechnology, Cat# C0017) according to the manufacturer’s instructions. The absorbance value was read at 490 nm. The death rate was calculated using the following formula: cell death rate % = (absorbance_sample_ – absorbance_control_)/(absorbance_max_ – absorbance_control_) × 100.

### Statistical analyses

No statistical methods were used to predetermine sample sizes; however, our sample sizes are similar to those reported in previous publications (Gong and Wei, 2025). At least three independent replicates were performed for each experiment. Data were analyzed statistically and visualized using GraphPad Prism (version 8.4.2, GraphPad Software, Inc., San Diego, CA, USA). All data are expressed as mean ± standard deviation. Data were compared using a two-tailed Student’s *t*-test or one-way analysis of variance with Tukey’s *post hoc* test. For all analyses, *P* < 0.05 was considered statistically significant.

## Results

### Murine double minute 2 expression is increased by oxygen and glucose deprivation combined with hemin, and its knockdown inhibits oxygen and glucose deprivation combined with hemin-induced ferroptosis and M1/M2 polarization in BV2 microglial cells

**Figure 1:**
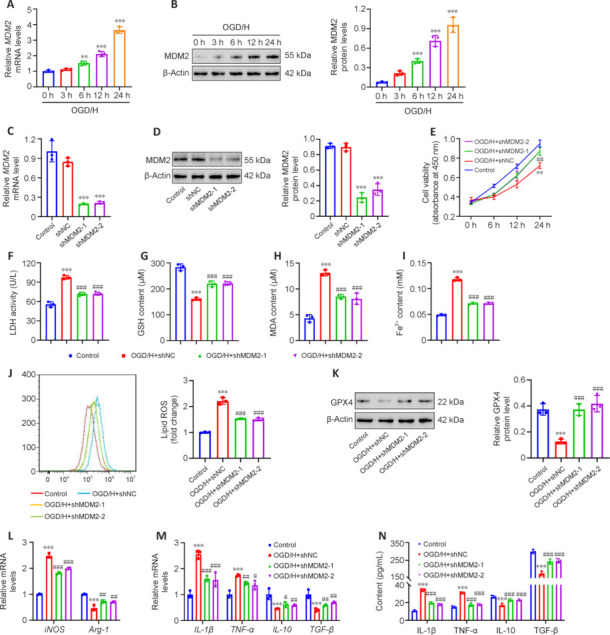
MDM2 knockdown inhibits OGD/H-induced ferroptosis and M1/M2 polarization in BV2 microglial cells. (A) mRNA and (B) protein expression of MDM2 in BV2 microglial cells after OGD/H treatment for 0, 3, 6, 12, and 24 hours. (C) mRNA and (D) protein expression of MDM2 in BV2 microglial cells transduced with the shMDM2-1, shMDM2-2, or shNC lentivirus vector. (E) BV2 microglial cells were pretransduced with shMDM2-1, shMDM2-2, or shNC lentivirus vector for 24 hours, followed by OGD/H treatment for 0, 3, 6, 12, and 24 hours. Cell viability was measured using the Cell Counting Kit-8 assay. (F–N) BV2 microglial cells were pretransduced with shMDM2-1, shMDM2-2, or shNC lentivirus vector for 24 hours, followed by OGD/H treatment for 24 hours. (F) LDH activity, (G) GSH content, (H) MDA content, (I) Fe^2+^ content, (J) lipid ROS levels, (K) GPX4 protein expression, (L) mRNA expression of *iNOS* and *Arg-1*, (M) mRNA expression of *IL-1*β, *TNF-α*, *IL-10*, and *TGF-*β, and (N) release of IL-1β, TNF-α, IL-10 and TGF-β were measured. Cells transduced with shNC lentivirus vector without OGD/H treatment were used as the control. All data are presented as the mean ± SD (*n* = 3). **P < 0.01, ***P < 0.001, *vs.* 0 h or control; #*P* < 0.05, ##*P* < 0.01, ###*P* < 0.001, *vs*. OGD/H + shNC (one-way analysis of variance with Tukey’s *post hoc* test). Arg-1: Arginase 1; GPX4: glutathione peroxidase 4; GSH: glutathione; IL-1: interleukin-1; IL-10: interleukin-10; iNOS: inducible nitric oxide synthase; LDH: lactate dehydrogenase; MDA: malondialdehyde; MDM2: murine double minute 2; OGD/H: oxygen–glucose deprivation combined with hemin; ROS: reactive oxygen species; scramble shRNA: shNC; TGF-β: transforming growth factor-β; TNF-α: tumor necrosis factor-α.

MDM2 is a vital oncogene associated with regulation of the cell cycle and apoptosis (Zafar et al., 2023), and it has significant effects in neurological disorders (Xu et al., 2018; Guo et al., 2021). We initially examined MDM2 expression in BV2 microglial cells subjected to OGD/H for varying durations. As shown in **Figure 1A** and **B**, MDM2 expression significantly increased with prolonged OGD/H treatment.

To investigate the effect of MDM2 on ICH, BV2 microglial cells were transduced with MDM2-interfering RNA (shMDM2-1 and shMDM2-2) or shNC lentivirus vector, and the cell viability and LDH activity were measured. qRT-PCR and western blotting confirmed successful MDM2 knockdown in the indicated cells (**Figure 1C** and **D**). After OGD/H treatment, cell viability significantly decreased and LDH activity increased. By contrast, shMDM2-1 or shMDM2-2 lentivirus vector transduction significantly enhanced cell viability and reduced LDH activity (**Figure 1E** and **F**). Several cell death inhibitors were evaluated to determine the cause of OGD/H- or MDM2-induced cell death. The ferroptosis inhibitor Fer-1 markedly inhibited OGD/H- or MDM2-induced cell death, whereas the apoptosis inhibitor Z-VAD-FMK only mildly suppressed OGD/H-induced cell death and marginally inhibited MDM2-induced cell death. The necroptosis inhibitor necrostatin-1 had no effect on OGD/H- or MDM2-induced cell death (**Additional Figure 1A** and **B**). These findings suggested that the cell death induced by OGD/H or MDM2 is primarily due to ferroptosis.

To further examine the effects of MDM2 on OGD/H-mediated ferroptosis, GSH and MDA content, GPX4 expression, Fe^2+^ ion content, and lipid ROS levels were measured. As shown in **Figure 1G–K**, OGD/H reduced GSH, increased MDA, Fe^2+^, and lipid ROS levels, and inhibited GPX4 expression. Furthermore, OGD/H increased mRNA expression of M1-type markers inducible nitric oxide synthase (*iNOS*), *IL-1*β, and *TNF-α*, and inhibited mRNA expression of M2-type markers arginase 1 (*Arg-1*), *IL-10*, and *TGF-*β (**Figure 1L** and **M**). Moreover, OGD/H increased TNF-α and IL-1β production and suppressed IL-10 and TGF- production (**Figure 1N**), indicating a role in regulating inflammatory responses. These effects were reversed with shMDM2-1 or shMDM2-2 lentivirus vector transduction (**Figure 1G–N**). Furthermore, Fer-1 treatment inhibited OGD/H- induced M1 polarization and inflammatory responses in BV2 microglial cells (**Additional Figure 2A** and **B**). These results indicated that MDM2 knockdown inhibits OGD/H-induced ferroptosis and inflammatory responses in BV2 microglial cells.

**Figure 2:**
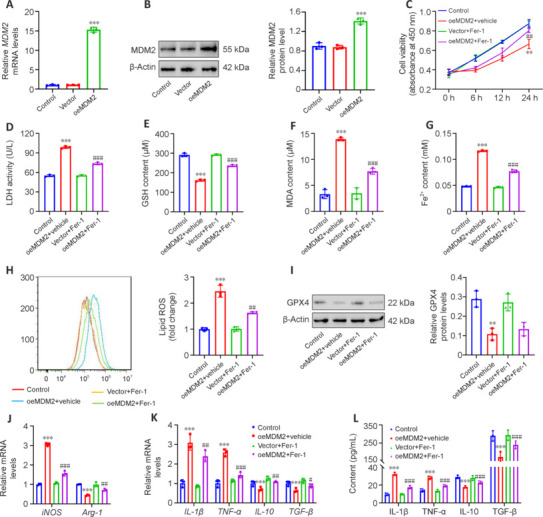
MDM2 overexpression induces M1/M2 polarization in BV2 microglial cells via ferroptosis. (A) mRNA and (B) protein expression of MDM2 in BV2 microglial cells transduced with the oeMDM2 lentivirus vector. (C–L) BV2 microglial cells were pretransduced with the oeMDM2 or blank lentivirus vector for 24 hours, followed by Fer-1 or vehicle treatment for 24 hours. (C) Cell viability using the Cell Counting Kit-8 assay, (D) LDH activity, (E) GSH content, (F) MDA content, (G) Fe^2+^ content, (H) lipid ROS, (I) GPX4 protein expression, (J) mRNA expression of *iNOS* and *Arg-1*, (K) mRNA expression of *IL-1*β, *TNF-α*, *IL-10*, and *TGF-*β, and (L) release of IL-1β, TNF-α, IL-10, and TGF-β were measured. Cells transduced with the blank lentivirus vector and treated with the vehicle were used as the control. All data are presented as the mean ± SD (*n* = 3). **P < 0.01, ***P < 0.001, *vs*. control; #*P* < 0.05, ##*P* < 0.01, ###*P* < 0.001, *vs.* oeMDM2 + vehicle (one-way analysis of variance with Tukey’s *post hoc* test). Arg-1: Arginase 1; GPX4: glutathione peroxidase 4; GSH: glutathione; IL-1: interleukin-1 beta; IL-10: interleukin-10; iNOS: inducible nitric oxide synthase; LDH: lactate dehydrogenase; MDA: malondialdehyde; MDM2: murine double minute 2; OD: optical density; ROS: reactive oxygen species; TGF-β: transforming growth factor-β; TNF-α: tumor necrosis factor-α.

### Murine double minute 2 overexpression induces M1/M2 polarization via ferroptosis

To investigate the effect of ferroptosis on MDM2-mediated inflammatory responses, BV2 microglial cells were pretransduced with an oeMDM2 lentivirus vector for 24 hours, followed by Fer-1 treatment for 24 hours. qRT-PCR and western blotting confirmed successful MDM2 overexpression (**Figure 2A** and **B**). Compared with the control group, MDM2 overexpression significantly reduced cell viability and increased LDH activity, whereas Fer-1 treatment significantly enhanced cell viability and decreased LDH activity (**Figure 2C** and **D**). MDM2 overexpression significantly reduced GSH, increased MDA, Fe^2+^, and lipid ROS levels, and inhibited GPX4 expression (**Figure 2E–I**). Moreover, MDM2 overexpression promoted M1 polarization, inhibited M2 polarization, and increased the inflammatory response (**Figure 2J–L**). These effects were reversed by Fer-1 treatment (**Figure 2C–L**). These results indicated that MDM2 overexpression induces inflammatory responses in BV2 microglial cells via ferroptosis-associated mechanisms.

### Murine double minute 2 induces glutathione peroxidase 4 ubiquitination and degradation

**Figure 3:**
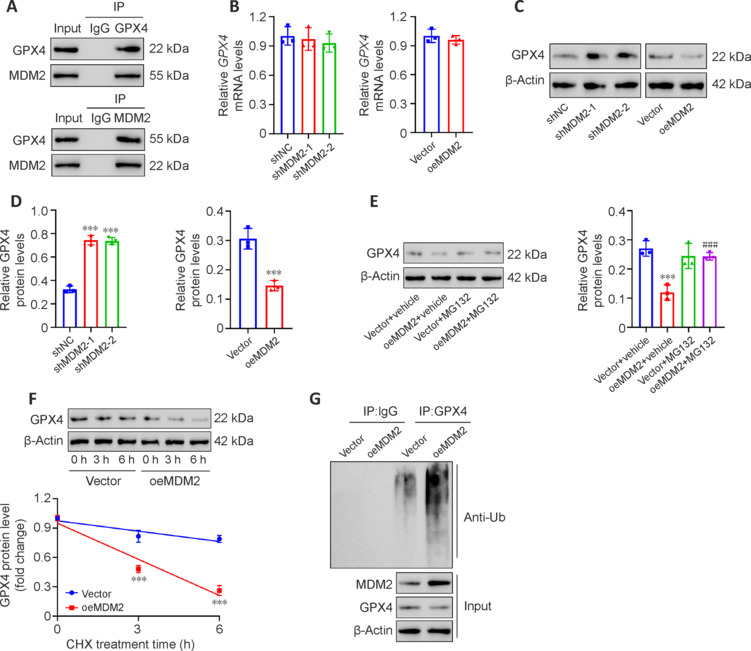
MDM2 induces GPX4 ubiquitination and degradation. (A) BV2 microglial cell lysates were subjected to immunoprecipitation with control IgG, anti-MDM2, or anti-GPX4 antibody. The immunoprecipitants were subsequently blotted with the indicated antibodies. (B) mRNA and (C, D) protein expression of GPX4 were measured in BV2 microglial cells transduced with shMDM2-1, shMDM2-2, shNC, oeMDM2, or blank lentivirus vector. (E) BV2 microglial cells transduced with the oeMDM2 or blank lentivirus vector were treated with MG132 or vehicle, and GPX4 protein expression was measured. (F) BV2 microglial cells transduced with the oeMDM2 or blank lentivirus vector were treated with CHX, and GPX4 protein expression was measured. (G) BV2 microglial cells were transduced with the oeMDM2 or blank lentivirus vector, and GPX4 was immunoprecipitated and immunoblotted with the indicated antibodies. All data are presented as the mean ± SD (*n* = 3). ***P < 0.001, *vs*. shNC, vector, or vector + vehicle; ###*P* < 0.001, *vs.* oeMDM2 + vehicle. (B right, D right, and F) Student’s *t*-test and (B left, D left, and E) one-way analysis of variance and the Tukey’s *post hoc* test were used. CHX: Cycloheximide; GPX4: glutathione peroxidase 4; IgG: immunoglobin G; IP: immunoprecipitation; MDM2: murine double minute 2.

To determine the molecular mechanism by which MDM2 increases GPX4 protein expression, a coimmunoprecipitation assay was performed, which indicated that MDM2 specifically bound to GPX4 protein (**Figure 3A**). MDM2 is an E3 ubiquitin ligase that regulates ubiquitination by binding specifically to the substrate, followed by proteasomal degradation. Therefore, we hypothesized that MDM2 regulates GPX4 protein levels. As shown in **Figure 3B–D**, MDM2 downregulation increased GPX4 protein levels, whereas MDM2 overexpression decreased them, with no effects on GPX4 mRNA levels. This decrease in GPX4 protein level was reversed by adding proteasome inhibitor MG132, suggesting that MDM2 regulates GPX4 protein levels in a proteasome-dependent manner (**Figure 3E**). To further determine whether MDM2 regulates GPX4 protein stability, cells were treated with CHX, and the half-life of GPX4 was determined. As shown in **Figure 3F**, GPX4 protein stability was significantly decreased in MDM2-overexpressed cells. These findings indicated that MDM2 destabilizes GPX4 in BV2 microglial cells. Next, we assessed whether MDM2 regulates GPX4 ubiquitination in cells. As shown in **Figure 3G**, MDM2 overexpression significantly increased GPX4 ubiquitination. These results suggested that MDM2 ubiquitinates GPX4 in BV2 microglial cells.

### Murine double minute 2 regulates oxygen and glucose deprivation combined with hemin-induced ferroptosis and M1/M2 polarization by targeting glutathione peroxidase 4

**Figure 4:**
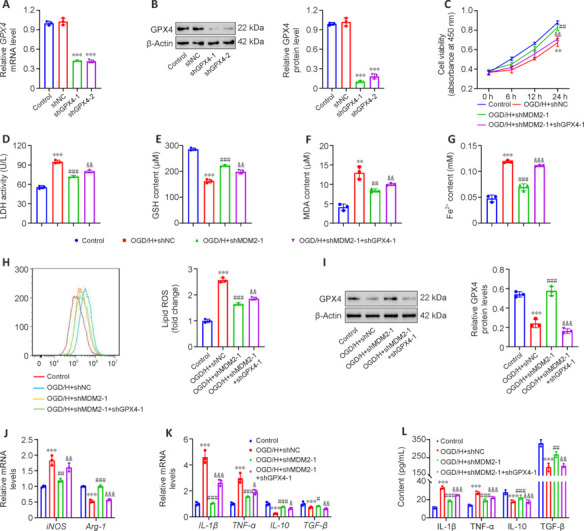
MDM2 regulates OGD/H-induced ferroptosis and M1/M2 polarization in BV2 microglial cells by targeting GPX4. (A) mRNA and (B) protein expression of GPX4 in BV2 microglial cells transduced with the shGPX4-1, shGPX4-2, or shNC lentivirus vector. (C) BV2 microglial cells were pretransduced with shMDM2-1, shGPX4-1, or shNC lentivirus vector for 24 hours, followed by OGD/H treatment for 0, 3, 6, 12, and 24 hours. Cell viability was measured using the Cell Counting Kit-8 assay. (D–L) BV2 microglial cells were pretransduced with shMDM2-1, shGPX4-1, or shNC lentivirus vector for 24 hours, followed by OGD/H treatment for 24 hours. (D) LDH activity, (E) GSH content, (F) MDA content, (G) Fe^2+^ content, (H) lipid ROS, (I) GPX4 protein expression, (J) mRNA expression of *iNOS* and *Arg-1*, (K) mRNA expression of *IL-1*β, *TNF-α*, *IL-10*, and *TGF-*β, and (L) release of IL-1β, TNF-α, IL-10, and TGF-β were measured. Cells transduced with shNC lentivirus vector and without OGD/H treatment were used as the control. All data are presented as the mean ± SD (*n* = 3). **P < 0.01, ***P < 0.001, *vs.* control; #*P* < 0.05, ##*P* < 0.01, ###*P* < 0.001, *vs.* OGD/H + shNC; &*P* < 0.05, &&*P* < 0.01, &&&*P* < 0.001, *vs*. OGD/H + shMDM2-1 (one-way analysis of variance with Tukey’s *post hoc* test). Arg-1: Arginase 1; GPX4: glutathione peroxidase 4; GSH: glutathione; IL-1: interleukin-1; IL-10: interleukin-10; iNOS: inducible nitric oxide synthase; LDH: lactate dehydrogenase; MDA: malondialdehyde; MDM2: murine double minute 2; OD: optical density; OGD/H: oxygen–glucose deprivation combined with hemin; ROS: reactive oxygen species; scramble shRNA: shNC; TGF-β: transforming growth factor-β; TNF-α: tumor necrosis factor-α.

To investigate the role of GPX4 in MDM2-mediated ferroptosis and inflammatory responses in OGD/H-induced BV2 microglial cells, BV2 microglial cells were pretransduced with shMDM2-1 and shGPX4-1 lentivirus vectors for 24 hours, followed by OGD/H treatment for 24 hours. qRT-PCR and western blotting confirmed that MDM2 and GPX4 were successfully knocked down (**Figure 4A** and **B**). MDM2 knockdown significantly enhanced the OGD/H-treated BV2 microglial cell viability, reduced LDH activity, increased GSH, decreased MDA, Fe^2+^, and lipid ROS levels, and increased GPX4 expression, which were reversed by GPX4 knockdown (**Figure 4C–I**). Furthermore, shMDM2-1 lentivirus vector transduction inhibited M1 polarization, promoted M2 polarization, and inhibited the inflammatory response in OGD/H-induced BV2 microglial cells (**Figure 4J–L**), and shGPX4 lentivirus vector transduction reversed the effects of shMDM2 (**Figure 4C–L**). These results indicated that MDM2 regulates OGD/H-induced ferroptosis and inflammatory responses in BV2 microglial cells by targeting GPX4.

### Wilms tumor 1-associated protein induces murine double minute 2 m6A modification

**Figure 5:**
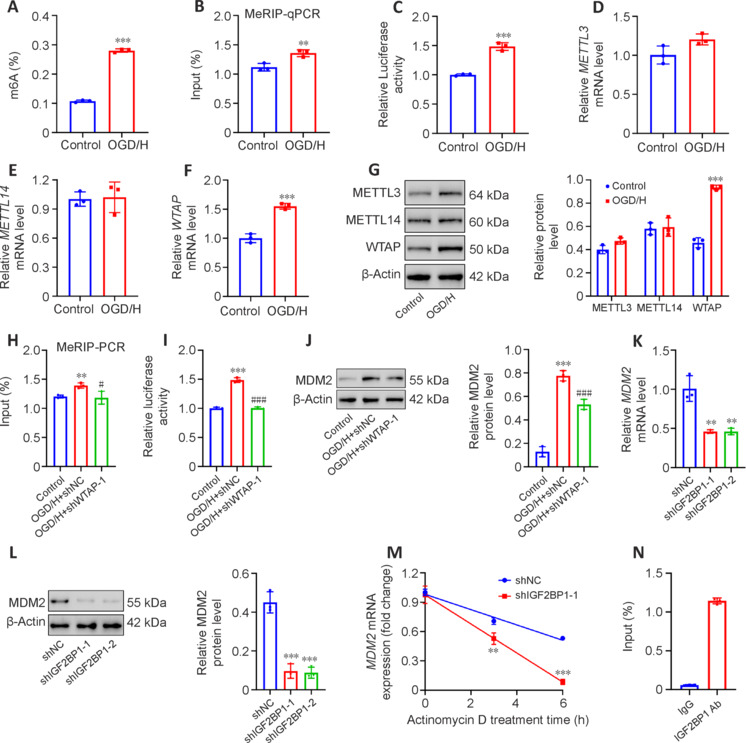
WTAP induces MDM2 m6A modification. (A) Global m6A, (B) m6A, and (C) luciferase activity of MDM2 3′ UTR, (D–F) mRNA and (G) protein expression of METTL3, METTL14, and WTAP in BV2 microglial cells were measured after OGD/H treatment for 24 hours. Cells without OGD/H treatment were used as the control. BV2 microglial cells were pretransduced with shWTAP-1 or shNC lentivirus vector for 24 hours, and (H) m6A and (I) luciferase activity of MDM2 3′ UTR and (J) MDM2 protein expression were measured after OGD/H treatment for 24 hours. Cells transduced with shNC lentivirus vector and without OGD/H treatment were used as the control. BV2 microglial cells were transduced with shIGF2BP1-1, shIGF2BP1-2, or shNC lentivirus vector for 48 hours, and the (K) mRNA and (L) protein expression of MDM2 were measured. (M) BV2 microglial cells transduced with shIGF2BP1-1 or shNC lentivirus vector were treated with actinomycin D, and *MDM2* mRNA expression was measured. (N) Interaction between IGF2BP1 and MDM2 3′ UTR (RNA immunoprecipitation assay). All data are presented as the mean ± SD (*n* = 3). **P < 0.01, ***P < 0.001, *vs.* control or shNC; #*P* < 0.05, ###*P* < 0.001, *vs*. OGD/H + shNC. (A–G and M) Student’s *t*-test and (H–L) one-way analysis of variance with Tukey’s *post hoc* test. IGF2BP1: Insulin-like growth factor 2 mRNA binding protein 1; m6A: N6-methyladenosine; MDM2: murine double minute 2; MeRIP-qPCR: m6A methylated RNA immunoprecipitation quantitative polymerase chain reaction; METTL: methyltransferase like; OGD/H: oxygen–glucose deprivation combined with hemin; UTR: untranslated region; WTAP: Wilms tumor 1-associated protein.

m6A modification is the most abundant posttranscriptional modification that regulates RNA stability, splicing, processing, transcription, and translation (Jiang et al., 2021). RNA-binding proteins, such as IGF2BPs, have been reported to enhance the stability and expression of MDM2 mRNA in an m6A-dependent manner (Mu et al., 2022; Han et al., 2024). We next investigated whether m6A modification regulates MDM2 mRNA stability and expression in OGD/H-induced BV2 microglial cells. After 24 hours of OGD/H treatment, global m6A levels were increased (**Figure 5A**). OGD/H significantly increased m6A levels and enhanced MDM2 3′ UTR luciferase activity (**Figure 5B** and **C**). OGD/H treatment had no significant effect on METTL3 and METTL14 expression in BV2 microglial cells; however, it significantly increased WTAP expression (**Figure 5D–G**). qRT-PCR and western blotting indicated that WTAP and IGF2BP1 were successfully knocked down in BV2 microglial cells (**Additional Figure 3A–D**). Furthermore, shWTAP-1 lentivirus vector transduction significantly decreased m6A levels and the luciferase activity of MDM2 3′ UTR, and inhibited MDM2 expression (**Figure 5H–J**). Similarly, shIGF2BP1-1 or shIGF2BP1-2 lentivirus vector transduction markedly suppressed MDM2 expression (**Figure 5K** and **L**). To elucidate the mechanism by which IGF2BP1 regulates MDM2 expression, BV2 microglial cells were treated with the transcriptional inhibitor actinomycin D. shIGF2BP1-1 lentivirus vector transduction accelerated MDM2 mRNA degradation (**Figure 5M**). The RIP-PCR results showed that IGF2BP1 specifically bound to MDM2 3′ UTR (**Figure 5N**). These results indicated that WTAP induces MDM2 m6A modification and that IGF2BP1 enhances MDM2 mRNA stability and expression.

### Wilms tumor 1-associated protein-mediated murine double minute 2 m6A modification regulates oxygen and glucose deprivation combined with hemin-induced ferroptosis and M1/M2 polarization in BV2 microglial cells

**Figure 6:**
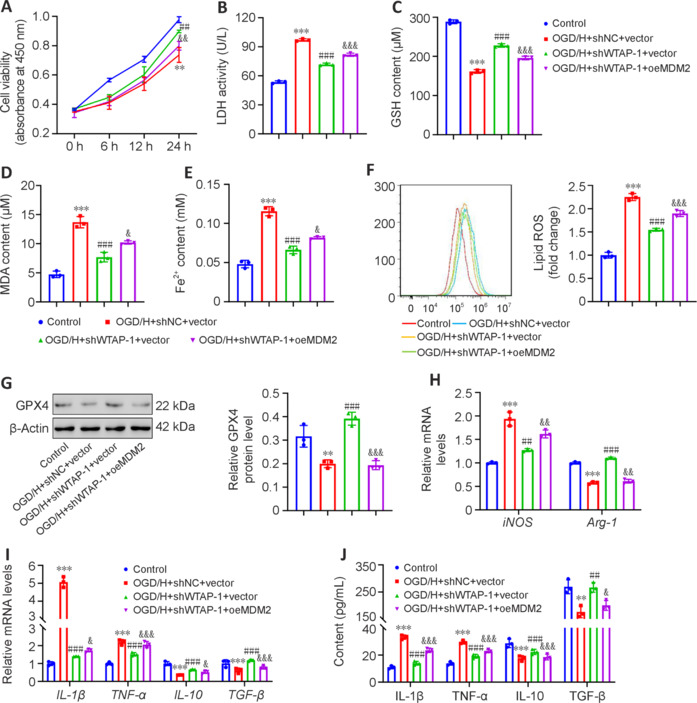
WTAP-mediated MDM2 m6A modification regulates OGD/H-induced ferroptosis and M1/M2 polarization in BV2 microglial cells. (A) BV2 microglial cells were pretransduced with shWTAP-1, shNC, oeMDM2, and blank lentivirus vector for 24 hours, followed by OGD/H treatment for 0, 3, 6, 12, and 24 hours. Cell viability was measured using the Cell Counting Kit-8 assay. (B–J) BV2 microglial cells were pretransduced with shWTAP-1, shNC, oeMDM2, and blank lentivirus vector for 24 hours, followed by OGD/H treatment for 24 hours. (B) LDH activity, (C) GSH content, (D) MDA content, (E) Fe^2+^ content, (F) lipid ROS, (G) GPX4 protein expression, (H) mRNA expression of *iNOS* and *Arg-1*, (I) mRNA expression of *IL-1*β, *TNF-α*, *IL-10*, and *TGF-*β, and (J) release of IL-1β, TNF-α, IL-10, and TGF-β were measured. Cells transduced with shNC lentivirus vector and the blank lentivirus vector, without OGD/H treatment were used as the control. All data are presented as the mean ± SD (*n* = 3). **P < 0.01, ***P < 0.001, *vs.* control; ##*P* < 0.01, ###*P* < 0.001, *vs*. OGD/H + shNC; &*P* < 0.05, &&*P* < 0.01, &&&*P* < 0.001, *vs*. OGD/H + shWTAP (one-way analysis of variance with Tukey’s *post hoc* test). Arg-1: Arginase 1; GPX4: glutathione peroxidase 4; GSH: glutathione; IL-1: interleukin-1; IL-10: interleukin-10; iNOS: inducible nitric oxide synthase; LDH: lactate dehydrogenase; MDA: malondialdehyde; MDM2: murine double minute 2; OD: optical density; OGD/H: oxygen–glucose deprivation combined with hemin; ROS: reactive oxygen species; scramble shRNA: shNC; TGF-β: transforming growth factor-β; TNF-α: tumor necrosis factor-α; WTAP: Wilms tumor 1-associated protein.

To investigate the effect of WTAP-mediated MDM2 m6A modification on OGD/H-induced ferroptosis and inflammatory responses, BV2 microglial cells were pretransduced with shWTAP-1, shNC, oeMDM2, or blank lentivirus vectors for 24 hours, followed by OGD/H treatment for 24 hours. Compared with the OGD/H + shNC group, the OGD/H + shWTAP-1 group had significantly improved BV2 microglial cell viability and decreased LDH activity, which were reversed by MDM2 overexpression (**Figure 6A** and **B**). Furthermore, shWTAP-1 lentivirus vector transduction significantly increased GSH, decreased MDA, lipid ROS, and Fe^2+^ levels, and increased GPX4 expression in OGD/H-induced BV2 microglial cells (**Figure 6C–G**). WTAP-mediated m6A modification of MDM2 also increased GPX4 ubiquitination in OGD/H-induced BV2 microglial cells (**Additional Figure 4**). As shown in **Figure 6H–J**, shWTAP-1 lentivirus vector transduction significantly inhibited M1 polarization, promoted M2 polarization, and inhibited the inflammatory response. However, combined transduction with an oeMDM2 lentivirus vector reversed the aforementioned effects of shWTAP-1 lentivirus vector transduction (**Figure 6C–J**). These results indicated that the WTAP-mediated m6A modification of MDM2 regulates OGD/H-induced ferroptosis and inflammatory responses in BV2 microglial cells.

### Murine double minute 2 inhibitor attenuates impaired neurofunction in mice with intracerebral hemorrhage by regulating the murine double minute 2–glutathione peroxidase 4 regulatory axis

**Figure 7:**
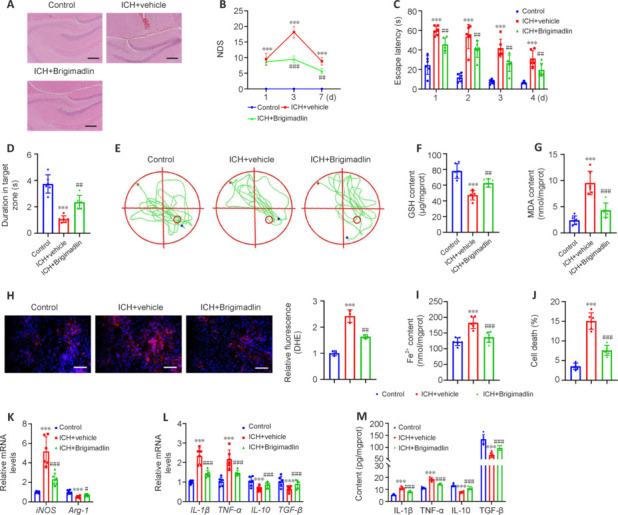
Brigimadlin attenuates spatial memory impairment in mice with ICH. ICH mice were treated with the MDM2 inhibitor brigimadlin or vehicle and then assessed for (A) HE staining (scale bars: 200 μm), (B) NDS, (C) escape latency, (D) time spent in the target zone, (E) swimming paths in the Morris water maze test, (F) GSH content, (G) MDA content, (H) DHE staining (scale bars: 100 μm), (I) Fe^2+^ content, (J) cell death, (K) mRNA expression of *iNOS* and *Arg-1*, (L) mRNA expression of *IL-1*β, *TNF-α*, *IL-10*, and *TGF-*β, and (M) release of IL-1β, TNF-α, IL-10, and TGF-β in brain tissue. Mice with sham surgery and treated with the vehicle were used as the control. All data are presented as the mean ± SD (*n* = 3 or 6). ***P < 0.001 *vs.* control; #*P* < 0.05, ##*P* < 0.01, ###*P* < 0.001 *vs*. ICH + vehicle (one-way analysis of variance with Tukey’s *post hoc* test). Arg-1: Arginase 1; DHE: dihydroethidium; GSH: glutathione; HE: hematoxylin and eosin; ICH: intracerebral hemorrhage; IL-1: interleukin-1; IL-10: interleukin-10; iNOS: inducible nitric oxide synthase; MDA: malondialdehyde; NDS: neurological deficit score; TGF-β: transforming growth factor-β; TNF-α: tumor necrosis factor-α.

To examine the effect of MDM2 on ICH *in vivo*, an ICH mouse model was established, and MDM2 inhibitor brigimadlin or vehicle was administered. HE staining showed that, compared with the control group, the ICH + vehicle group had hematoma lesions and inflammatory cell infiltration (**Figure 7A**). In the behavioral tests, the ICH + vehicle group had increased neurological deficit scores and escape latency and decreased duration in the target zone (**Figure 7B–E**). The ICH + vehicle group also had decreased GSH levels and increased MDA, Fe^2+^, and ROS levels, cell death rate, and inflammatory response compared with those in the control group (**Figure 7F–M**). All of these data indicated successful establishment of the ICH model. Treatment with MDM2 inhibitor brigimadlin reversed the aforementioned effects of ICH (**Figure 7A–M**).

**Figure 8:**
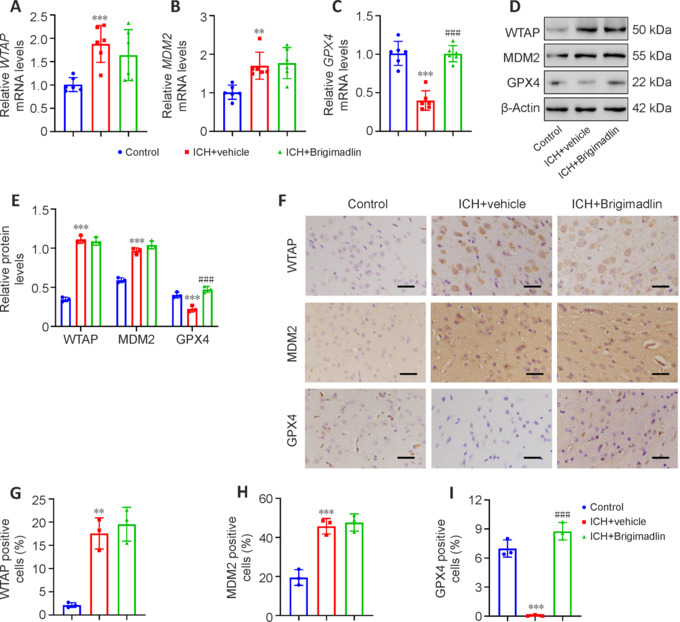
WTAP, MDM2, and GPX4 expression in mice with ICH. (A–C) mRNA expression of *WTAP*, *MDM2*, and *GPX4* in brain tissue of ICH mice treated with the MDM2 inhibitor brigimadlin or vehicle, measured using quantitative reverse transcription-polymerase chain reaction. (D, E) Western blotting and (F–I) immunohistochemistry staining (scale bars: 25 μm) detected the protein expression of WTAP, MDM2, and GPX4 in brain tissue of ICH mice treated with the MDM2 inhibitor brigimadlin or vehicle. Mice with sham surgery and treated with the vehicle were used as the control. All data are presented as the mean ± SD (*n* = 3 or 6). **P < 0.01, ***P < 0.001, *vs.* control; ##*P* < 0.01, ###*P* < 0.001 *vs*. ICH + vehicle (one-way analysis of variance with Tukey’s *post hoc* test). GPX4: Glutathione peroxidase 4; ICH: intracerebral hemorrhage; MDM2: murine double minute 2; WTAP: Wilms tumor 1-associated protein.

ICH significantly increased mRNA and protein levels of WTAP and MDM2 and decreased GPX4 mRNA and protein levels. Brigimadlin treatment restored GPX4 expression and had no effect on WTAP and MDM2 expression (**Figure 8A–E**). Immunohistochemical analysis showed that brigimadlin increased GPX4 expression and did not affect WTAP and MDM2 expression (**Figure 8F–I**). These results indicated that MDM2-induced GPX4 ubiquitination serves as a critical mediator in neurological impairment in the pathogenesis in ICH, and is related to ferroptosis and oxidative stress.

## Discussion

It has been shown that m6A RNA modification facilitates tumorigenesis and tumor progression, but its role in ICH is poorly understood. Microglia-driven neuroinflammation contributes to brain damage following ischemia and reperfusion, and m6A modification has been implicated in this process (Yu et al., 2023). Following ICH onset, brain tissue damage occurs via both hematoma-induced direct mechanical compression and ischemia-hypoxia, which directly affects prognosis and treatment outcomes (Chen et al., 2021). Therefore, in the present study, BV2 microglial cells were subjected to OGD/H to mimic ICH *in vitro*. OGD/H treatment induces PC12 cell apoptosis (Chen et al., 2021) and ferroptosis and the inflammatory response of brain microvascular endothelial cells (Lu et al., 2020; Jiang et al., 2022). The findings of this study indicate that OGD/H treatment induced ferroptosis, promoted M1 polarization and inflammatory response, and increased MDM2 protein expression in BV2 microglial cells, which subsequently downregulated GPX4 protein expression by increasing its ubiquitination, resulting in OGD/H-induced microglial dysfunction.

As the primary components of brain microvasculature, microglia play important roles in maintaining normal physiological function. Microglia respond to acute brain injury by activating M1- (proinflammatory) or M2-type (anti-inflammatory) cytokines and chemokines (Lan et al., 2017). Lei et al. (2023) demonstrated that preventing M1 microglial polarization inhibited microglial cell pyroptosis and decreased neuroinflammation after cerebral hemorrhage, suggesting that microglia are essential in ICH pathology and recovery. This study used OGD/H-treated BV2 microglial cells *in vitro* to explore neuronal function. Programmed cell death, particularly apoptosis, participates in early cerebral damage following ICH. Ferroptosis is a distinct iron-dependent cell death pathway driven by lipid peroxidation. Suppressing neuronal cell apoptosis and ferroptosis via the p53–SLC7A11–GPX4 regulatory axis protects against ICH (Yang et al., 2025). Additionally, hemolysis during ICH produces iron ions that attack DNA, protein, and lipid membranes by inducing hydroxyl radical formation, thus resulting in neuronal apoptosis (Gong et al., 2024). These findings suggest that apoptosis and ferroptosis may interact during cell death. Our investigation showed that OGD/H treatment decreased cell viability, which was inhibited by apoptosis and ferroptosis inhibitors, with a higher inhibitory effect by the ferroptosis inhibitor. These results suggested that OGD/H-induced cell death is primarily due to ferroptosis.

A previous study indicated the cytotoxic role of MDM2 in brain tissue damage. MDM2 activated p53 to induce brain microvascular endothelial cell (BMVEC) apoptosis, promoting ischemic stroke (Zhang et al., 2018). Furthermore, MDM2 stabilized the E2F1 protein by repressing its ubiquitination, contributing BMVEC dysfunction and brain injury after ICH (Guo et al., 2021). Our results showed increased MDM2 protein levels in ICH models, and interfering with MDM2 inhibited ferroptosis and the inflammatory response, indicating its cytotoxic role in ICH. In line with our findings, MDM2 was shown to cause mitochondrial respiratory chain damage, produce excessive mitochondrial ROS and autophagosomes, and induce inflammation in BV2 microglial cells (Zhang et al., 2023). However, MDM2 expression in brain tissues peaks at 24 hours post-ICH, and the MDM2-mediated p53 pathway has been shown to protect against ICH-induced neuronal apoptosis (Xu et al., 2018; Barrio et al., 2021). Furthermore, MDM2 upregulation inhibited p53 expression and protected against ROS-dependent neuronal apoptosis (Zhao et al., 2020). These contrasting roles may result from the heterogeneity of cell death and inflammatory responses exhibited by MDM2 in post-ICH and BV2 microglial cells. Notably, the present study found that OGD/H and ICH promoted p53 protein expression and acetylation modification (data not shown), inducing downstream p53-responsive genes. p53 acetylation inhibits its MDM2-mediated ubiquitination and enhances DNA-binding activity, increasing the activity and expression of p53 protein (Li et al., 2002). Furthermore, apoptosis may also contribute to MDM2-induced neurotoxicity via cell death-associated inhibitors.

GPX4 is a multifunctional antioxidant enzyme that uses reduced GSH levels as a cofactor to detoxify lipid peroxidation and inhibits ferroptosis (Miao et al., 2022). In this study, MDM2 overexpression suppressed GPX4 expression, induced lipid peroxidation, and increased Fe^2+^. A previous study showed that as an E3 ubiquitin ligase, MDM2 mediating PSD-95 ubiquitination and degradation increased infarct volume and neuronal apoptosis in mice with cerebral ischemia (Lv et al., 2021). Moreover, MDM2 led to p53 ubiquitination, activating LMNB1 and consequently aggravating mitochondrial damage and ferroptosis in kidney tubular epithelial cells after acute kidney injury (Hu et al., 2023). In the present study, MDM2 was identified as a binding partner of GPX4, and MDM2 increased GPX4 ubiquitination and degradation, leading to ferroptosis. Overexpression of the ferroptosis-related gene *ACSL4* was shown to induce ferroptosis and M2-to-M1 macrophage polarization (Chen et al., 2023). In this study, GPX4 knockdown increased the expression of M1-type markers (iNOS, TNF-α, and IL-1β) and inhibited M2-type markers (Arg-1, IL-10, and TGF-β) in OGD/H-induced BV2 microglial cells. Furthermore, a ferroptosis inhibitor attenuated the inhibitory effects of MDM2 overexpression on BV2 microglial cell polarization. Collectively, these results indicated that MDM2-induced ferroptosis mediated M1/M2 polarization in OGD/H-induced BV2 microglia.

m6A is a common chemical modification of human mRNA that serves as a crucial mechanism in neurological disorders such as Parkinson’s and Alzheimer’s diseases (Liu et al., 2024). Our results indicated that WTAP impairment inhibited ferroptosis and M1/M2 polarization in OGD/H-induced BV2 microglial cells by targeting MDM2. m6A exerts its gene-regulatory function via recognition by m6A reader proteins. Several m6A readers participate in ICH progression by controlling mRNA stability (Khan et al., 2024). IGF2BP1 contributes to cell proliferation and growth in various diseases, including tumors and Alzheimer’s disease (Sun et al., 2024; Yao et al., 2024). Our findings showed that IGF2BP1 binds to the 3′ UTR of MDM2 and regulates its mRNA stability *in vitro*.

This study was limited by the absence of *in vivo* validation of the upstream regulatory role of WTAP in ferroptosis and inflammation in mice with ICH. Additionally, primary cultured microglia could be beneficial for further *in vitro* studies. Other potential upstream posttranscriptional or posttranslational modifications regulating MDM2 expression remain unclear. Finally, whether pyroptosis and apoptosis are involved in MDM2-induced cytotoxicity in ICH remains unknown. These limitations will be addressed in our following study.

In conclusion, the current study provides *in vitro* and *in vivo* data demonstrating that MDM2 silencing alleviates ICH-induced neurological dysfunction and OGD/H-induced brain microglial cell injury, highlighting its potential application for treating ICH-induced brain damage. Furthermore, MDM2 downregulated GPX4 protein levels by facilitating its ubiquitination, contributing to OGD/H-induced microglial dysfunction. These findings suggest that targeting MDM2 and GPX4 may offer a promising therapeutic strategy for ICH in the future.

## Additional files:

***Additional Figure 1:*** *Effects of Fer-1, Z-VAD-FMK, or necrostatin-1 on OGD/H or MDM2-induced cell death.*

Additional Figure 1 Effects of Fer-1, Z-VAD-FMK, or necrostatin-1 on OGD/H or MDM2-induced cell death.
(A) Cell viability of BV2 microglial cells treated with OGD/H for 24 hours, followed by Fer-1, Z-VAD-FMK, necrostatin-1, or vehicle treatment for 24 hours. Cells with vehicle and without OGD/H treatment were used as the control. (B) BV2 microglial cells were pretransduced with oeMDM2 or blank lentivirus vector for 24 hours, followed by Fer-1, Z-VAD-FMK, necrostatin-1, or vehicle treatment for 24 hours. Cell viability was measured by Cell Counting Kit-8 assay. Cells transduced with blank lentivirus vector and treated with vehicle were used as the control. All data are presented as the mean ± SD (*n* = 3). ^***^P <0.001, *vs*. control; ^#^*P* < 0.05, ^##^*P* < 0.01, ^###^*P* < 0.001, *vs*. OGD/H + vehicle or oeMDM2 + vehicle (one-way analysis of variance with Tukey's *post hoc* test). Fer-1: Ferrostatin-1; MDM2: murine double minute 2; OD: optical density; OGD/H: oxygen–glucose deprivation combined with hemin.

***Additional Figure 2:*** *Fer-1 inhibits OGD/H-induced M1/M2 polarization and inflammatory responses in BV2 microglial cells.*

Additional Figure 2 Fer-1 inhibits OGD/H-induced M1/M2 polarization and inflammatory responses in BV2 microglial cells.
BV2 microglial cells were treated with Fer-1 for 24 hours, followed by OGD/H treatment for 24 hours. (A) mRNA expression of iNOS and Arg-1 and (B) release of IL-1β, TNF-α, IL-10, and TGF-β were measured. Cells without OGD/H and Fer-1 treatment were used as the control. All data are presented as the mean ± SD (*n* = 3). ^***^P < 0.001, *vs*. control; ^#^*P* <0.05, ^##^*P* < 0.01, ^###^*P* < 0.001, *vs*. OGD/H (one-way analysis of variance with Tukey's *post hoc* test). Arg-1: Arginase 1; Fer-1: ferrostatin-1; IL-1β: interleukin-1β; IL-10: interleukin-10; iNOS: inducible nitric oxide synthase; OGD/H: oxygen–glucose deprivation combined with hemin; TGF-β: transforming growth factor-β; TNF-β: tumor necrosis factor-β.

***Additional Figure 3:*** *WTAP and IGF2BP1 expression in BV2 microglial cells.*

Additional Figure 3 WTAP and IGF2BP1 expression in BV2 microglial cells.
mRNA and protein expression of (A, B) WTAP2 and (C, D) IGF2BP1 in BV2 microglial cells transduced with shWTAP2-1, shWTAP2-2, shIGF2BP1-1, shIGF2BP1-2, or shNC lentivirus vector. All data are presented as the mean ± SD (*n* = 3). ^***^P < 0.001, *vs*. shNC (one-way analysis of variance with Tukey's *post hoc* test). IGF2BP1: Insulin-like growth factor 2 mRNA binding protein 1; scramble shRNA: shNC; WTAP: Wilms tumor 1-associated protein.

***Additional Figure 4:*** *WTAP-mediated m6A modification of MDM2 promotes ubiquitination of GPX4.*

Additional Figure 4 WTAP-mediated m6A modification of MDM2 promotes ubiquitination of GPX4.
BV2 microglial cells were pretransduced with shWTAP, shNC, oeMDM2, and blank lentivirus vector for 24 hours, followed by OGD/H treatment for 24 hours. GPX4 was immunoprecipitated and immunoblotted with the indicated antibodies. Cells transduced with shNC lentivirus vector and blank lentivirus vector, without OGD/H treatment were used as control. GPX4: Glutathione peroxidase 4; IP: immunoprecipitation; MDM2: murine double minute 2; OGD/H: oxygen–glucose deprivation combined with hemin; scramble shRNA: shNC; Ub: ubiquitin; WTAP: Wilms tumor 1-associated protein.

***Additional Table 1:*** *shRNA sequences used in the study.*

***Additional Table 2:*** *The primers used for quantitative reverse transcription-polymerase chain reaction analysis.*

## Data Availability

*All relevant data are within the paper and its Additional files*.
