# Supplementary figures and images for: MDM2–GPX4–ferroptosis regulatory axis exerts neurotoxic effects in intracerebral hemorrhage
Source: Neural Regen Res. 2025 Aug 13;21(7):3063–72. doi: 10.4103/NRR.NRR-D-25-00030 (PMC13378939; doi:10.4103/NRR.NRR-D-25-00030)

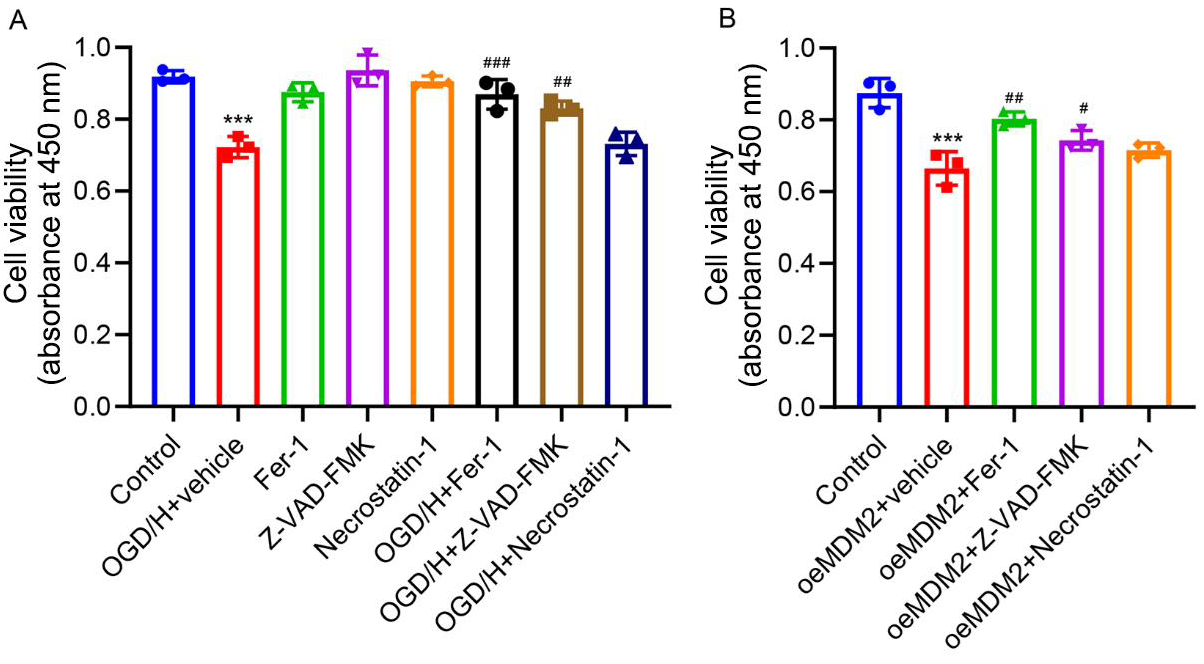

Supplement: Supplementary file 1 [file NRR-21-3063_Suppl1.tif]

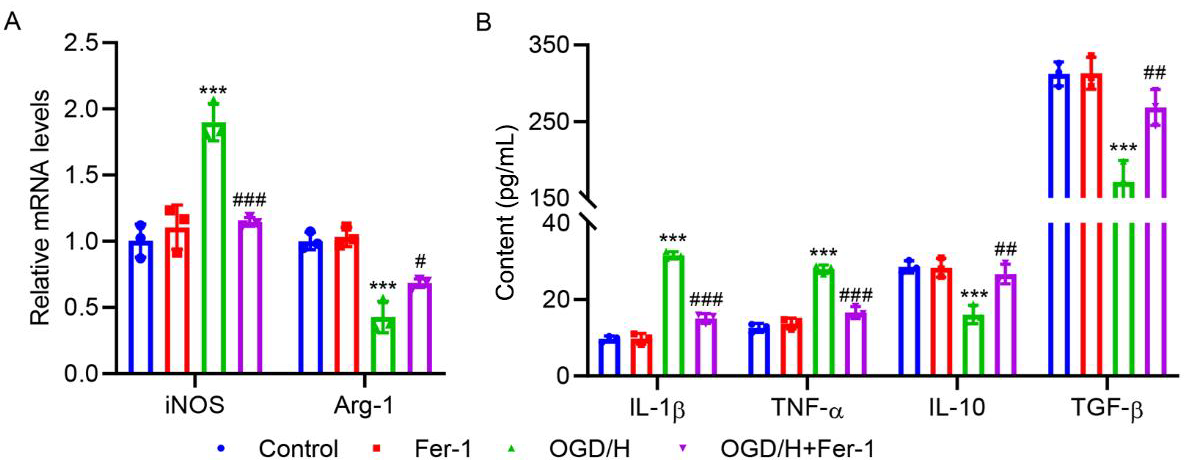

Supplement: Supplementary file 2 [file NRR-21-3063_Suppl2.tif]

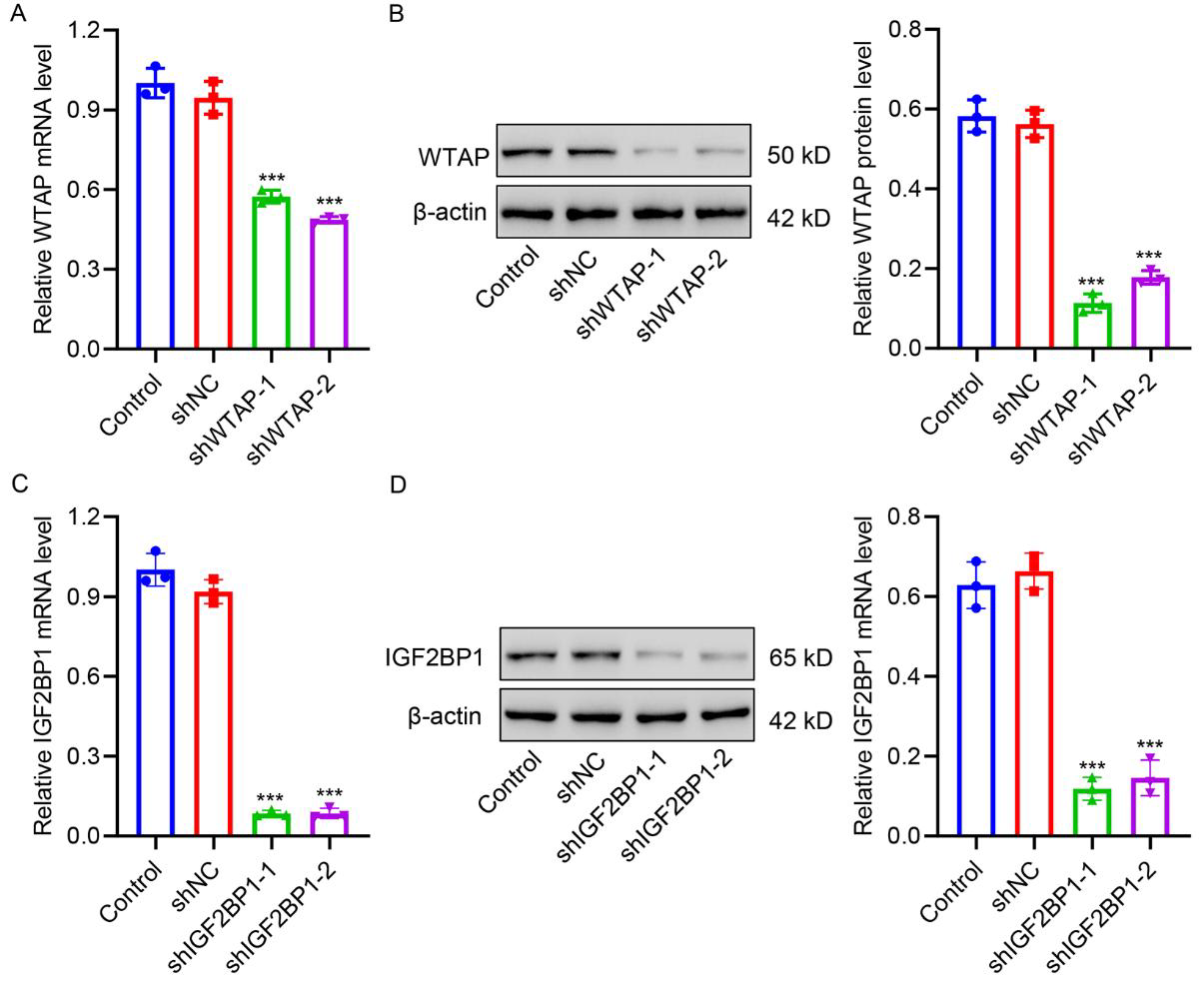

Supplement: Supplementary file 3 [file NRR-21-3063_Suppl3.tif]

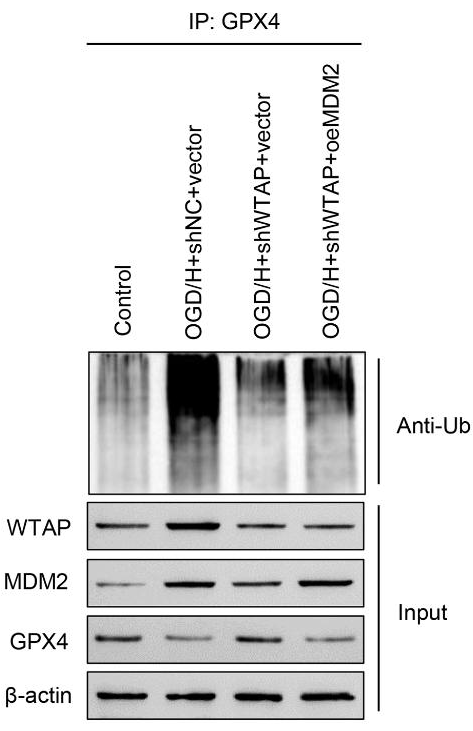

Supplement: Supplementary file 4 [file NRR-21-3063_Suppl4.tif]
